# Idiopathic Infected Hydrocele in a Toddler: A Case Report with Review

**DOI:** 10.1100/tsw.2006.371

**Published:** 2006-03-23

**Authors:** Jayesh Sagar, Suhas Kumar, D. Mondal, D.K. Shah

**Affiliations:** ^1^ Royal Free Hospital, London, UK; ^2^ S.S.G.Hospital, Vadodara, India

**Keywords:** idiopathic, infected hydrocele, toddler

## Abstract

Idiopathic infected hydrocele in infants is a rare, but well-documented, entity in English literature; however, occurrence of such a condition in a toddler is not yet documented. Here we report the case of an idiopathic infected hydrocele in a toddler for the first time in English literature. We also discuss a review of literature and demonstrate management of infected hydrocele by antibiotics without any surgical intervention, also for the first time in English literature.

## INTRODUCTION

Infected hydrocele in infants is a well-known entity in English literature. A few cases of the idiopathic nature of the infected hydrocele are also documented for infants. However, presentation of idiopathic infected hydrocele in toddlers has not been documented. We report the case of an idiopathic infected hydrocele in a 3-year-old boy who responded well to antibiotics without any surgical intervention. We emphasize consideration of this rare condition in differential diagnosis of scrotal swelling in toddlers. Here, we also suggest the trial of nonsurgical treatment with antibiotics before deciding on surgical intervention if certain of diagnosis.

## CASE HISTORY

A 3-year-old boy with painful right hemiscrotum was presented in the Accident and Emergency Department. The child complained of severe pain and had red, swollen, and tender right hemiscrotum. He denied any other complaints. Apart from the use of inhalers for his recent upper respiratory tract infection, he was fit and healthy. On examination, he had a temperature of 39.5°C. The vital signs were stable. Examination of external genitals revealed tender, swollen, red, and hot right side of scrotum. Due to pain, he did not allow further examination of the right testis. The left side of scrotum and testis were normal. The systemic examination did not reveal any positive findings. He underwent urgent color doppler ultrasonography of the scrotum, which suggested turbid fluid with fibrin in the hydrocele sac with normal blood flow and size of testicles. The urine examination did not show any positive findings. The blood results did not reveal any abnormality including WBC of $9.4 \times 10^3$ µl. The blood culture was sent for culture and sensitivity. The patient was admitted to the pediatrics ward under surgical care and was started on analgesia and intravenous augmentine. The patient responded well to antibiotics and recovered well on the 4th day of admission. The repeat ultrasonography revealed clear fluid surrounding normal right testis. The blood culture did not grow any organisms. The patient was discharged on the 5th day with follow-up arranged as an outpatient in a week's time.

## DISCUSSION

Hydrocele, the most common cause of scrotal swelling[1], is a fluid collection within the tunica vaginalis of the testis or along the spermatic cord. Infected hydrocele, although rare, is a well-documented entity in infants in English literature. The common causes of infected hydrocele in infants are muconium orchitis[2], epididymo-orchitis secondary to infection elsewhere in the body such as urinary tract infection[3] or from maternal puerperal sepsis[4], incarcerated inguinal hernia, testicular torsion, and scrotal hematoma[5]. There has been report of infected hydrocele complicating acute appendicitis[6]. There are very few reported cases of idiopathic infected hydrocele in infants[7,8,9]. The most common causes of scrotal swelling in children in the western world include testicular torsion, incarcerated inguinal hernia, epididymo-orchitis, and idiopathic scrotal edema. The infected hydrocele is not well known in children, especially in toddlers, causes being secondary to sepsis[10] or as a complication of surgery including renal transplantation[11]. However, we should not forget filariasis as one of the most common causes of scrotal swelling in children in tropical countries[12]. Here we present a case of idiopathic infected hydrocele in a toddler. To our knowledge, such a case has not been reported in English literature.

The etiology of infected hydrocele remains unclear. The different theories include direct transmission of infection from epididymis, testicles or one of its appendages[10,13], hematogenous spread of infection[14], and seeding of infection from the peritoneal cavity through patent processus vaginalis[15,16]. There have been reports of infected hydrocele following appendectomy (the patients were 14 and 20 years old)[17,18]. In our case, we could not find any preliminary cause for infected hydrocele.

There are no distinguished clinical features pertaining to the infected hydrocele. As with other common conditions such as testicular torsion, incarcerated inguinal hernia, and epididymo-orchitis, acutely swollen, tender, erythematous, and irreducible scrotal mass with fever is the common presentation. As the testicular torsion and incarcerated inguinal hernia are emergent surgical conditions requiring immediate surgical interventions, these must be excluded before considering infected hydrocele. Because the idiopathic scrotal edema is painless and nontender, it can be differentiated from other acute scrotal conditions. Color doppler ultrasound is the investigation of choice as it would differentiate infected hydrocele from other pathologies. Presence of turbid fluid with fibrin material in the hydrocele sac suggests the diagnosis of infected hydrocele[7]. According to the literature, all the cases of infected hydrocele have undergone surgical intervention. The most common explanation is uncertainty of diagnosis, especially in infants, as it is difficult to perform sonography in infants. The other explanation may be late presentation. In our case, due to certainty of diagnosis, we decided to give a trial of intravenous antibiotics with very close observation without embarking on any surgical intervention. We think the early diagnosis was a favoring factor in the management of our case. However, we strongly advise surgical exploration if diagnosis is in doubt. As conservative management of infected hydrocele has not been documented in the literature before, it is very difficult to say that the infected hydrocele would resolve itself or not after conservative treatment, though in our case, the patient would need further repair of the hydrocele as it did not resolve after conservative treatment. One can argue on the basis of the just-mentioned statement why we should not operate/aspirate and get culture of infected fluid at the time of presentation rather than giving a trial of conservative treatment. We quite agree with this, however, we think it may be beneficial to operate on a hydrocele as a clean, elective surgical procedure rather than an emergency operation. Due to the rarity of this condition, at present it is very difficult to answer all the questions.

Here, we stalwartly suggest considering infected hydrocele in the differential diagnosis of acute scrotal swellings in toddlers. We also propose a trial of nonsurgical treatment in the form of intravenous antibiotics in confirmed cases, although if in doubt, we favor the surgical exploration of the scrotum.
